# Antibiotic-Resistant *Acinetobacter baumannii* in Low-Income Countries (2000–2020): Twenty-One Years and Still below the Radar, Is It Not There or Can They Not Afford to Look for It?

**DOI:** 10.3390/antibiotics10070764

**Published:** 2021-06-23

**Authors:** Soha S. Rizk, Wafaa H. Elwakil, Ahmed S. Attia

**Affiliations:** 1 Microbiology and Immunology Postgraduate Program, Faculty of Pharmacy, Cairo University, Cairo 11562, Egypt; soha.razk@std.pharma.cu.edu.eg (S.S.R.); wafaa.h.elwakil@std.pharma.cu.edu.eg (W.H.E.); 2 Department of Microbiology and Immunology, Faculty of Pharmacy, Cairo University, Cairo 11562, Egypt

**Keywords:** *A. baumannii*, low-income, surveillance, developing countries, carbapenem-resistance, MDR, Syria, Ethiopia, COVID-19

## Abstract

*Acinetobacter baumannii* is an emerging pathogen, and over the last three decades it has proven to be particularly difficult to treat by healthcare services. It is now regarded as a formidable infectious agent with a genetic setup for prompt development of resistance to most of the available antimicrobial agents. Yet, it is noticed that there is a gap in the literature covering this pathogen especially in countries with limited resources. In this review, we provide a comprehensive updated overview of the available data about *A. baumannii*, the multi-drug resistant (MDR) phenotype spread, carbapenem-resistance, and the associated genetic resistance determinants in low-income countries (LIICs) since the beginning of the 21st century. The coverage included three major databases; PubMed, Scopus, and Web of Science. Only 52 studies were found to be relevant covering only 18 out of the 29 countries included in the LIC group. Studies about two countries, Syria and Ethiopia, contributed ~40% of the studies. Overall, the survey revealed a wide spread of MDR and alarming carbapenem-resistance profiles. Yet, the total number of studies is still very low compared to those reported about countries with larger economies. Accordingly, a discussion about possible reasons and recommendations to address the issue is presented. In conclusion, our analyses indicated that the reported studies of *A. baumannii* in the LICs is far below the expected numbers based on the prevailing circumstances in these countries. Lack of proper surveillance systems due to inadequate financial resources could be a major contributor to these findings.

## 1. Introduction

The ceaseless emergence of new pathogens and the constant mutations into pathogenic resistant strains are today’s humanity’s most tremendous threats. Carbapenem-resistant *Acinetobacter baumannii* (CRAP) is currently ranked first on the World Health Organization list of critical pathogens urgently in need of new antibiotic development. What is considered a low-virulence pathogen with reduced adhesion and invasion abilities has now surfaced as a hard to contain agent with the genetic setup for prompt development of resistance mechanisms to most of the available antimicrobial agents [1]. *Acinetobacter* was first isolated in 1911 from the soil [2]. It underwent taxonomic changes from its primary designated name *Micrococcus calcoaceticus* until it received the genus *Acinetobacter* due to its nonmotile nature. It is an aerobic Gram-negative, catalase-positive, oxidase-negative, nonfermenting coccobacillus belonging to the Moraxellacea family with a DNA G+C content ranging from 39% to 47% [2,3]. Being a non-lactose-fermenter with the ability to survive on low nutritional requirements enabled its germination in a wide range of environments. Moreover, its ability to form biofilms, resist dissection, and persist for extended periods of time on surfaces, especially in hospital settings, led to multiple outbreaks [4,5,6].

*A. baumannii* causes too many types of infections including ventilator-associated pneumonia, meningitis, urinary tract infection, bone/wound infections, and the most serious type is the bacteremia. What makes *A. baumannii* more critical is that the mortality rates associated with it could reach up to 35% and escalate to 43% in intensive care units (ICUs) [7]. The global prevalence of *A. baumannii*, its widespread antibiotic resistance, and most recently, the unforeseen emergence and spread of clinical isolates resistant to colistin, the last resort in our current antimicrobial arsenal [8], all this necessitates a more prominent understanding of this challenging organism. The emphasis of the current review will be on the economically disadvantaged populations residing in low-income countries (LICs). It is challenging to portray an accurate picture of the spread of *A. baumannii* and its antibiotic susceptibility profile in different settings in this part of the world. Notably, there is a great disparity in the economic resources available in low-income countries and the rest of the world. Accordingly, this could be reflected on the detection capabilities and the level of details and sophistication of the reports about *A. baumannii* outbreaks. In this review, we present a comprehensive coverage of how *A. baumannii* and its resistance to antibiotics have been spreading across geographical borders throughout the LICs in the last 21 years since the beginning of the 21st century.

## 2. Methodology

The scientific literature in the three databases PubMed, Scopus, and Web of Science was screened in this order. The search terms included “*baumannii*” and the name of each of the twenty-nine countries included in the 2020/2021 World Bank classification and categorized as LICs [9]. The date limit was set to start from January 2000 to December 2020. In the case of a country name with any known variations, the different versions of the name were included in separate searches. No limit was imposed on the language with which the article was published. Accordingly, the studies included English, French, and Spanish articles. Both original reports and case reports were included.

**Figure 1:**
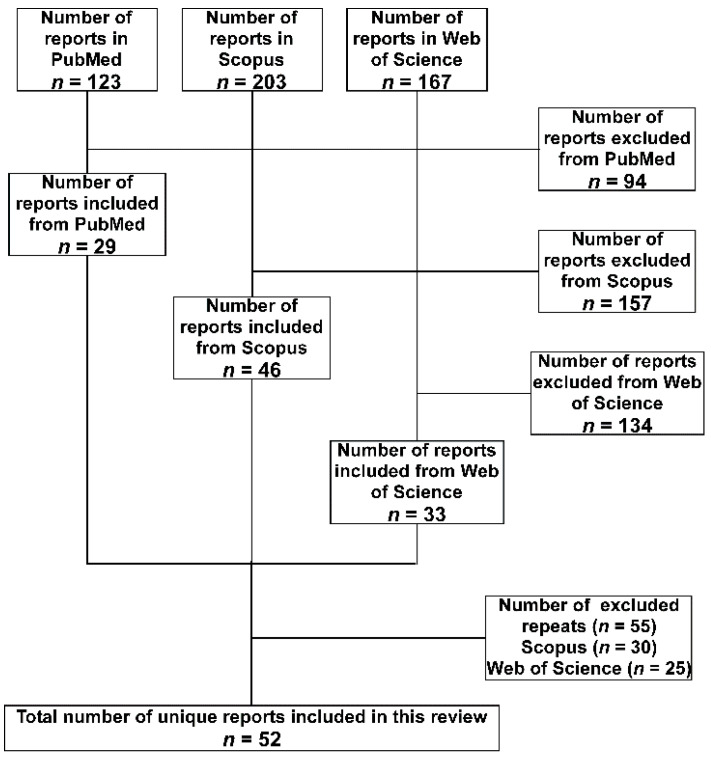
Flow diagram of the search strategy and selection of articles adopted in the current review. The databases were searched in the order (PubMed, Scopus, and Web of Science). Accordingly, articles found in the subsequent database(s) were considered a repeat and excluded.

A total of 493 hits were obtained from the three databases, then the following inclusion criteria were applied to the retrieved studies to select the relevant ones. The study had to include the detection of *A. baumannii* isolate or its DNA either in a medical or an environmental setting. The isolate or the DNA should have been detected in a setting located within one of the 29 LICs included in the study. Furthermore, studies of isolates confirmed to be isolated from individuals who are nationals of the targeted LICs but were transferred to be treated in other countries, either for war conflicts or political instabilities, were also included. Studies focused on performing research on standard strains to test the efficacy of medications or natural products collected from the targeted LICs were excluded. Finally, studies with just one, or more, of the authors who have an affiliation associated with one of the 29 LICs, but without including isolates or nucleic acids that fit the above criteria, were excluded. Repeated studies among the database were excluded considering first the ones detected in PubMed, then Scopus, and finally Web of Science. A summary of the search methodology adopted in the current study is presented in Figure 1.

The total number of included studies after applying the exclusion criteria was 52. They were analyzed for the method of identification and any phenotypic or genotypic characterizations. Special attention was paid to the antibiotics susceptibility profiles and the detection of the multi-drug-resistance (MDR) which was defined as being resistant to at least three antibiotics belonging to different classes. If the study included testing of resistance to carbapenems, the percentage of resistance was also noted.

## 3. *A. baumannii* in the LICs Distributed over the Different Geographical Regions

The 29 LICs included in the World Bank classification are distributed over six geographical regions; Sub-Saharan Africa (*n* = 23), the Middle East and North Africa (*n* = 2), South Asia (*n* = 1), Latin America and The Caribbean (*n* = 1), Europe and Central Asia (*n* = 1), and East Asia and Pacific (*n* = 1). Accordingly, this review will address the studies that originated from each of these six regions. In particular, an overview of the incidences of *A. baumannii* infections, the MDR phenotype spread, the presence of carbapenem-resistance, the associated genetic resistance determinants if available, and any phenotypic or genotypic characterization performed.

### 3.1. Sub-Saharan Africa

The Sub-Saharan Africa region included a total of 23 countries of the LICs. The reports about *A. baumannii* in these countries in the searched databases are summarized below. They are arranged in a descending order according to the number of reports related to each country.

**Ethiopia** is ranked the first among this group in the number of reports about *A. baumannii* infections with a total of 10 studies. The first report of the detection of *A. baumannii* in this country came in 2012, and it was isolated from human head and body lice collected from healthy individuals [10]. The genotypic analysis of these isolates showed that these particularly sensitive strains harbored several hundred insertion sequence elements that served in their genome reduction (disruptions of genes and simple loss of DNA). Additionally, it was pointed out that they have low catabolic capacities compared to the human MDR *A. baumannii* isolates. This signifies the adaptation of this bacterium to the louse environment [10]. In the same year, *A. baumannii* was reported in human infections for the first time from the ulcers of leprosy patients [11]. Yet, only 20% of these isolates showed MDR phenotype. It took almost five years to detect MDR *A. baumannii* that are carbapenem-resistant harboring the *bla*_NDM-1_ resistance determinant. However, susceptibility testing indicated that they remain susceptible to both polymyxin and amikacin [12]. Genome sequencing confirmed that the isolates carried the *bla*_NDM-1_; however, they are distinct from the outbreak strains reported from neighboring countries like Kenya. This argued against the regional spread of the *bla*_NDM-1_-positive organism but rather implied the independent environmental dependent generation of the resistance [12]. Moreover, MDR *A. baumannii* was isolated from a hospital’s air setup implicating this troublesome pathogen in the widespread colonization of the hospital’s setting [13]. The prevalence of MDR and Extended Spectrum Beta-Lactamase (ESBL) producers are reaching alarming rates from the isolated specimens in Ethiopian hospitals [14,15,16]. In another study, 33% of the isolates from nosocomial infections were meropenem-resistant [17]. The situation got more complicated in other instances where the isolates turned out to be extensively drug resistant (XDR); resistant to at least one agent in all but two or fewer antimicrobial categories, pan-drug-resistant (PDR); resistant to all antibiotic classes [18,19].

With only five studies dealing with *A. baumannii* infections, **Madagascar** came next in the list of LIC nations in this geographical sub-region. The first reports about *A. baumannii* outbreaks in Madagascar hospitals came in 2010 [20,21]. The isolated strains showed up to 44% carbapenem-resistance, and harboring genes encoding the β-lactamases AmpC, OXA23, and OXA51 [21]. Interestingly, the emergence of these strains in Madagascar preceded the availability of carbapenems in this country’s hospitals. Nosocomial infections between 2006 and 2013 were caused by carbapenem-resistant *A. baumannii* carrying only the *bla*_OXA23_ and *bla*_OXA51_ genes as the only carbapenemase-producing genes [21]. During the period between 2011 and 2013, MDR *A. baumannii* started to show up as one of the causative agents of urinary tract infections (UTI) in Madagascar; however, they were still carbapenem-sensitive [22]. On the other hand, reports about clinical samples collected between 2013 and 2016 indicated the predominance of Sequence Type 2 (ST2) and the acquisition of carbapenemases *bla*_OXA24_ and *bla*_OXA58_ for the first time since the first outbreak of *A. baumannii in* Madagascar [23]. In 2019, *A. baumannii* were detected in human head lice, yet they were negative for *bla*_OXA23_, *bla*_OXA24_, *bla*_IMP_, and *bla*_VIM_ [24].

Four reports have come from **Uganda** in the last 21 years reporting the detection of *A. baumannii* in the hospitals of this country. A recent report regarded *A. baumannii* as a minor cause of sepsis (~1%) in a multisite study [25]. An earlier report, examining the prevalence of *A. baumannii* and its rates of resistance between 2007 and 2009, showed that it was responsible for about 3% of the infections with 31% of these isolates carbapenem-resistant [26]. What was alarming in this report is that *A. baumannii* represented 14% of the environmental samples in the hospital and 55% were carbapenem-resistant. The β-lactamases encoding genes detected in this study included; *bla*_OXA23-like_, *bla*_OXA24-like_, *bla*_OXA58-like_, and *bla*_VIM-like_ [26]. Focusing on the isolates from the same hospital, cluster analysis using repetitive element sequence-based polymerase chain reaction (Rep-PCR) fingerprinting indicated a high level of genetic diversity among the isolates [27]. Yet, certain MDR isolates from the environment and patients were clustered, indicating possible environmental transmission of these strains to the patients in the hospital. Ten years later and in a follow-up study published in 2019, the same hospital reported a significant decrease in carbapenem resistance prevalence in the isolated *Acinetobacter* reaching only 2.7% [28]. However, all the carbapenemase-producing isolates were MDR and the *bla*_VIM_ was the most prevalent carbapenemase-encoding gene. An explanation for this decrease was not discussed by the authors, however a possible one could be the very limited number of the *A. baumannii* strains investigated in the earlier study as compared to the very large number of isolates in the later study (more than 1000). Accordingly, the high rates in the earlier study could be an over-estimation of the situation then.

Only two reports about *A. baumannii* infections in each of Burkina Faso, Democratic Republic of the Congo, Malawi, Mozambique, and Sudan were published in the last 21 years. For **Burkina Faso**, the first report came in 2016 about oral infections and they detected 3 *A. baumannii* strains out of 125 clinical samples [29]. All three were MDR and ESBL-positive and the β-lactamases encoding genes identified were *bla*_TEM_ and *bla*_CTX-M_. In a more recent study, few *A. baumannii* strains were isolated from a hospital there and they belonged to sequence type 2, or what is known as international clone II which has a high distribution worldwide [30,31]. On the other hand, in the **Democratic Republic of the Congo** (formally known as Zaire), a single report about nosocomial infections caused by *A. baumannii* came out in 2017 [32]. The isolates were not MDR and remained sensitive to both cefotaxime and gentamycin. Another report came from the same country in 2019; however, this time *A. baumannii* was isolated from human body lice but without determination of their antibiotic susceptibility patterns [33]. Regarding **Malawi**, a study published in 2012 reported the isolation of only one *A. baumannii* strain from a blood-stream infection of an HIV patient [34]. Following the incidence of bloodstream infections in children ≤5 years in a central hospital in Malawi in 20 years from 1998 to 2017, *A. baumannii* increased 10-fold from 0.2% (2003–2007) to 2.2% (2013–2017), and the isolates showed MDR phenotypes [35]. In **Mozambique**, a case of a hospitalized fatal pneumonia caused by an MDR *A. baumannii* strain belonging to the international clone II took place in 2014, yet it was not reported until 2018 as a case study [36]. The same case was reported earlier by another study in 2016 dealing with evaluating minimally invasive autopsy procedures to identify the infectious cause of death in the same hospital [37]. Finally, for **Sudan**, which has moved recently to the LIC group, the first report of *A. baumannii* was published in 2019 about the whole genome sequencing of an *A. baumannii* strain isolated from a hospitalized patient in the capital, Khartoum [38]. The sequence analyses indicated that the strain belonged to sequence type 164. In 2020, a survey of antimicrobial resistance in a hospital in Khartoum indicated that *A. baumannii* was the most frequent carbapenemase-producing organism (89% of the isolates) and they demonstrated higher resistance rates (100%) for cephalosporins and trimethoprim/sulfamethoxazole [39].

Only two studies about *A. baumannii* in **Rwanda** were published with the first one in 2004 and reported ten strains, from three different genotypes, detected in human body louse [40]. However, a very recent report came in 2020 and indicated the detection of *A. baumannii* in flies from a tertiary hospital [41]. Only one strain of *A. baumannii* was detected in this setting, yet its genome carried genes encoding virulence factors that are known to be associated with serum survival and invasion [41]. Only one study about *A. baumannii* in **Burundi** was published in 2004, which is the same report as the one mentioned above from the neighboring Rwanda. However, it only reported three strains detected in human body louse from the same genotype [40]. On the other hand, only one study reported *A. baumannii* infection in **Mali**, where it represented only 10% of the studied cases of pregnant women with cervicofacial cellulitis [42]. The isolated strain remained sensitive to amoxicillin/clavulanic acid. While in **Sierra Leone**, *A. baumannii* was responsible for 14 out of 164 nosocomial infections in an urban tertiary hospital [43]. The isolated strains were MDR and positive for ESBL, yet the carbapenem-resistance was as low as 10%.

There is only one report that originated from Somalia and was published online in November 2020 that describes the detection of *A. baumannii* in a hospital in **Somalia** [44]. In this report, *A. baumannii* was the most prevalent pathogen that belonged to MDR and XDR patterns in 69.1% of the samples with isolates showing 100% resistance rates against beta-lactam, cephalosporins, fluoroquinolones, and carbapenems. Finally, only one report about *A. baumannii* in head lice and **Niger** was published in 2018 [45]. In this case, *A. baumannii* were not detected in head lice from people residing in Niger; however, they were refugees from this country in neighboring Algeria.

**Figure 2:**
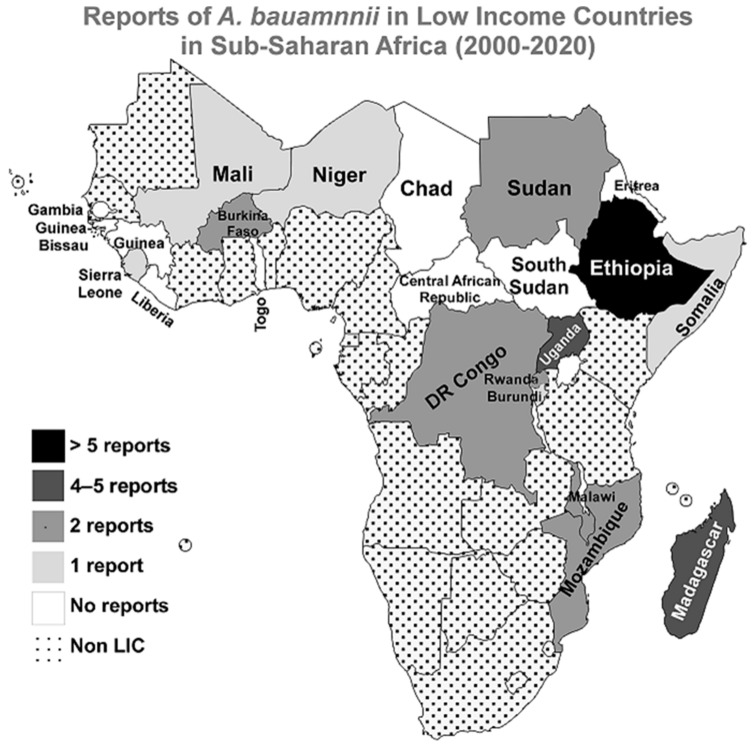
Reports of *A. baumannii* infections in LICs in Sub-Saharan Africa from 2000 to 2020. A geographical map of the Sub-Saharan Africa region highlighting the numbers of reports about *A. baumannii* infections in the 21 first years of the 21st century. The map was generated using the MapChart web tool (https://mapchart.net/, accessed on 21 June 2021).

No reports were published in the searched three databases for *A. baumannii* infections in the following countries since the beginning of the 21st century; **Central African Republic**, **Chad**, **Eritrea, Gambia**, **Guinea**, **Guinea-Bissau**, **Liberia**, **South Sudan**, and **Togo**. A graphical representation of the distribution of the included studies in the LICs in the Sub-Saharan region is presented in Figure 2.

### 3.2. Middle East and North Africa

Only two countries belonging to the LIC group are located within this geographical region: Syria and Yemen. Despite being ravaged by wars for many years within the last two decades, the linkage between **Syria** and *A. baumannii* infections has a very good share in the reports covered in this review; 10 studies. However, most of these reports were including Syrian patients who were treated in hospitals outside Syria especially in neighboring countries. The first report of *A. baumannii* in Syria was in 2012 and the study was conducted in a Syrian city showing high rates of MDR strains and the carbapenem-resistance was around 65% among them [46]. Yet, these strains remained susceptible to colistin. All the later reports dealt with Syrians treated in other countries. Wounded Syrians treated in Jordan showed up to 80% carbapenem-resistance among MDR *A. baumannii* infections between August 2011 and March 2013 [47]. Syrian bone-wounded civilians treated in Jordan and infected with *A. baumannii* were also among those reported in another study in 2017 together with wounded civilians from two other wars [48]. In another study analyzing data in Jordanian hospitals over a decade (2006–2016), 140 Syrians who had bone war wounds were included. This study showed that MDR *A. baumannii* was among the causative agents of these infections and the carbapenem-resistance level among these isolates was as high as 66.7% [49]. Moreover, Syrians treated in Israeli hospitals showed carriage of MDR *A. baumannii* [50]. Concerns about the exporting of NDM-1-producing *A. baumannii* strains and/or their genetic determinants from Syria was highlighted in a report from Turkey, where a Syrian refugee was linked to the introduction of the MDR and carbapenem-resistant strains to Turkey, and the strain belonged to sequence type 85 [51]. More recently in 2019, a Turkish study reported the detection of *A. baumannii* (~5%) among the causative agents of infection in Syrian war-injured patients [52]. As in Turkey, Lebanon too linked the introduction of MDR NDM-1 producing *A. baumannii* strains to Syrians injured in the war and transferred to Lebanese hospitals for treatment [53]. Similar to the Turkish case, the isolates belonged to sequence type 85 showing that Syria could constitute a reservoir for these strains. Two more studies from Lebanon reported the detection of MDR *A. baumannii* strains originating in Syria with carbapenem-resistance rates ranging from 74% to 100% [54,55].

The second country of the LIC group in the Middle East and North Africa is **Yemen**, where the situation is not greatly different from Syria as the country was inflicted with war for many years during the last 21 years. Only one study was reported from inside a hospital in Yemen, where three MDR carbapenem-resistant isolates were investigated [56]. Genetic analyses indicated that they harbor the *bla*_OXA23-like_, 16S rRNA methylase *armA*, and the acetyltransferase *aac*(6′)-Ib genes. The three strains belonged to sequence type 2 (ST2) and they were still susceptible to colistin. Yemen was linked to *A. baumannii* infections in another study but not for people receiving healthcare inside the country. In these cases, they were bones war-wounded patients hospitalized in Jordan and when detected, *A. baumannii* was carbapenem-resistant [49].

### 3.3. South Asia

Only **Afghanistan** is located in this geographical area and belongs to the LIC group. A single report came out in 2011 about Afghani patients treated in a deployed American Military Hospital that reported the recovery of *Acinetobacter* spp., including *A. baumannii*, from both patients and the hospital environment. Many of the isolated strains had high rates of antimicrobial resistance [57].

### 3.4. Latin America and the Caribbean

Only **Haiti** is included in this sub-category and the literature contained few reports about *A. baumannii* infection among Haitian people. The first report dealt with *A. baumannii* isolates collected from patients who had been injured in the Haiti earthquake in 2010 and they were *bla*_CTX-M-15-_positive [58]. Another report came in 2012 dealt also with the 2010 earthquake disaster and reported one case infected with MDR *A. baumannii* among the 14 wound-infection patients who had purulent discharges included in this study [59]. A third report dealt with infections burn units in Haiti. In this unit, *A. baumannii* was responsible for 15% of the reported infections, the *A. baumannii* associated with blood infection among patients who did not survive to discharge was resistant to aminoglycosides, fluoroquinolones, third-generation cephalosporins, and also carbapenems [60]. In a surveillance study in an obstetrics emergency hospital and neonatal care unit conducted during 2016, *A. baumannii* was isolated from two women’s rectal swabs [61]. In a recent metagenomic study to assess the quality of surface water in Haiti, *A. baumannii* was estimated to represent 5% of *Acinetobacter* species and 1% of total relative abundance [62]. These findings indicate that this pathogen is present in the water and could potentially be responsible for a proportion of the human infections in this country.

### 3.5. Europe and Central Asia

Only **Tajikistan** is located in this area; however, no reports about *A. baumannii* in this country were found in our search of the three respective databases.

### 3.6. East Asia and Pacific

**Democratic People’s Republic of Korea** (also known as **North Korea**) is the only low-income country that belongs to this geographical area. There are no records in PubMed directly reporting the isolation of *A. baumannii* in North Korea. Although, in the review by Zarrilli and co-workers [63], they citied another study by Yum and colleagues [64]. However, careful examination of the latter study indicated that this was a report about isolates from South Korea rather than its northern counterpart. Accordingly, it is safe to say that there are no reported studies about the detection of the *A. baumannii* in North Korea in the included databases in the last 21 years.

A summary of all the analyzed studies is presented in Table 1.

## 4. Discussion

Looking into the data reported from the 29 countries in the LIC group about *A. baumannii* clearly shows that there are very few reports coming out of this group of countries. Out of the 29 countries, only 16 countries reported detections of *A. baumannii* within their territories at least once in the last 21 years. Moreover, reports came about *A. baumannii* detection among citizens of two more countries; however, they were located in other countries either as refugees or asylum seekers. Accordingly, 11 out of the 29 have no *A. baumannii* reports at all in the searched three databases. The country with the highest number of reports was Ethiopia with 10 reports. On the other hand, Syria has ten reports too, yet only one of them was about Syrians within the territories of their own country. The earliest report about *A. baumannii* in LICs came in 2004 yet it was dealing with detecting *A. baumannii* in lice. Yet, the first report dealing with human infections was in 2010 originating from Madagascar. Then the studies started to follow on during the second decade of the 21st century. Interestingly, the first report of *A. baumannii* infections in the reviewed LIC countries came more than twenty years after its emergence as a nosocomial pathogen in the late 1980s of the 20th century [65].

**Figure 3:**
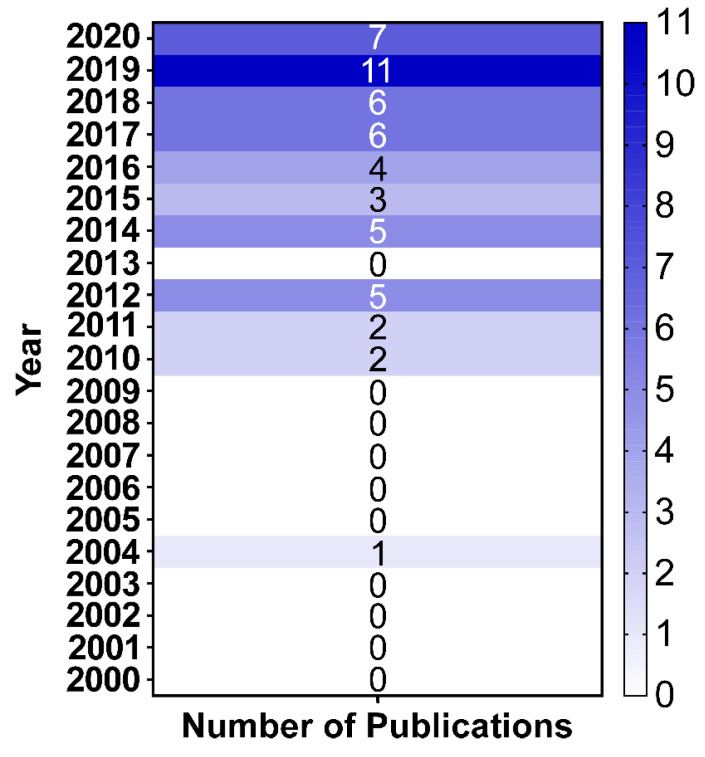
A heatmap of the distribution of the number of the publications about *A. baumannii* in LICs in the first 21 years of the 21st century. The heatmap was generated using GraphPad Prism v9 (GraphPad Software, San Diego, CA, USA).

Following the publication years of the reports, it was noted that starting from 2010 there was a gradual increase in the number of publications per year (Figure 3). Yet, there were no publications in 2013. The highest number of publications was recorded in 2019 with eleven studies. Those studies were linked to nine countries with one study covering patients from two countries: Syria and Yemen [49]. However, in 2020, we returned back to only seven studies. This decline could be attributed to the widespread of the COVID-19 pandemic through-out the world, which could have diverted some of the attention away from other pathogens such as the *A. baumannii*. This has been already reported in France raising the possibility that an outbreak could hide another one [66]. Of course, this should not exclude the possibility that the decline in reports about *A. baumannii* infections during the COVID-19 outbreak could be due to the increased understanding of sanitation and social distancing. Despite this observation, yet the list of publications about *A. baumannii* in LICs in 2020 showed the addition of new two countries for the first time: Sierra Leone and Somalia [43,44].

What is the situation of *A. baumannii* reports in countries with higher incomes? For instance, Nepal which was among the LICs in 2019 but moved to the middle-income countries (MIC) in 2020 has at least 19 reports of *A. baumannii* infections in the last 21 years. On the other hand, checking a country belonging to the high-income category like Germany reviewed by Wareth and co-workers, and applying stricter inclusion criteria than those applied here, found 44 studies (>84% of all the studies collectively included from LICs in the current review) in the period between 2000 and 2018 [67]. Another example from a country with a higher economy, Saudi Arabia, studies reported the detection of resistant *A. baumannii* from 2000 to 2015 reached up to 23 studies [68]. Could this disparity in the number of reports be a reflection of the actual real low incidence of *A. baumannii* in LICs as compared to countries with better economies? The answer to this question is most probably no. As within high-income countries themselves, the low socioeconomic status is a contributing risk factor for acquiring infections with antimicrobial resistant pathogens belonging to the ESKAPE group such as *Enterococcus faecalis*, *Escherichia coli*, *Klebsiella pneumoniae*, *Staphylococcus aureus*, and *A. baumannii* [69,70].

Since we are looking into *A. baumannii* in LICs, it was worth checking if it is the issue of detecting *A. baumannii* in this setting or it applies to other members of the ESCAPE pathogens. Checking reports about *K. pneumoniae* reports from Ethiopia (one of the two LICs with the highest numbers of *A. baumannii* reports), PubMed included at least 29 relevant studies from 2000 to 2020. This is almost triple the number of the *A. baumannii* reports in the three databases used in this review. Checking some of the countries, in the LICs group reporting no *A. baumannii* published studies in the searched databases, for *K. pneumoniae* related reports showed no reports either for North Korea, Tajikistan, Togo, and Liberia. However, Guinea-Bissau has at least two records [71,72], Central African Republic has at least one [73], and Chad has at least one too [74]. Accordingly, there could be some bias towards underreporting *A. baumannii* in LICs as compared to another resistant ESKAPE pathogen such as *K. pneumoniae*. Of course, one reason to explain this could be the fact that *K. pneumoniae* is a well-established pathogen known before *A. baumannii* took the stage in the field of clinical infections.

Looking into the number of the isolates investigated in the reviewed studies (Table 1), it is very clear that the numbers are very modest in many of the studies. The highest number of isolates investigated was in a study conducted in Uganda with 1077 isolates, yet the % of carbapenem-resistant isolates among them was just around 3% [28]. Accordingly, this could alert us that the high rates of carbapenem-resistance seen in the other studies (reaching 100% in some instances), could be just a reflection of the low numbers of isolates investigated in many of these studies. Accordingly, it could be very difficult to have a good estimate about the prevalence of carbapenem-resistance among the *A. baumannii* detected in the LICs.

**Figure 4:**
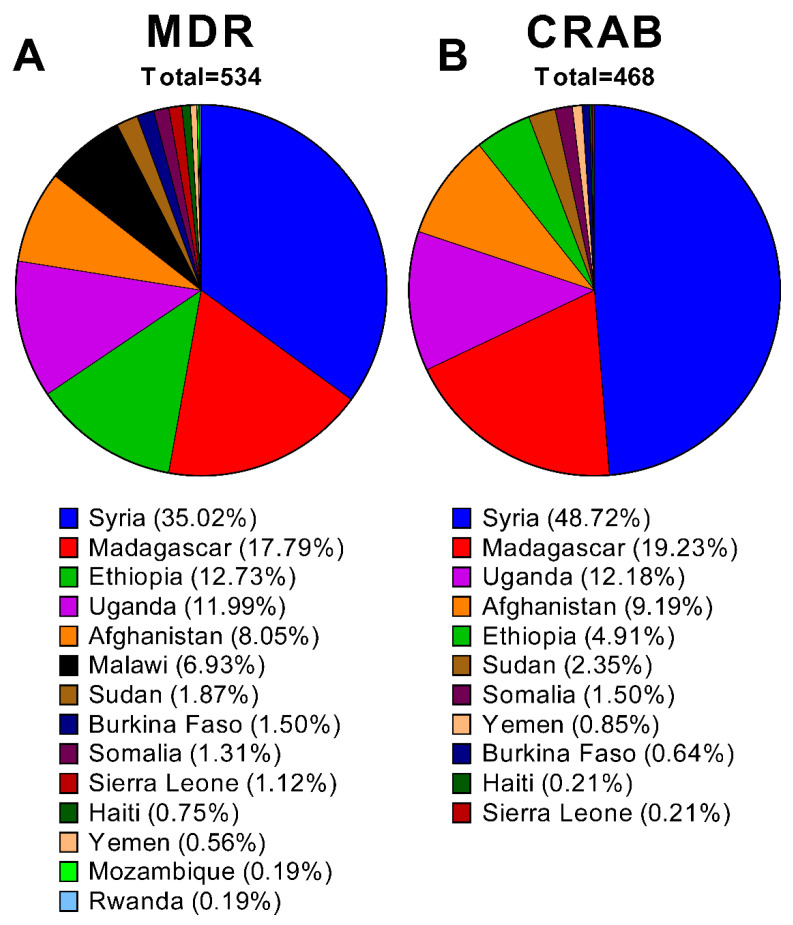
Pie charts of the contributions of the different LICs to the total number of MDR (**A**) and the CRAB (**B**) strains included in the reports analyzed in the current review. The percentages were calculated by dividing the number of strains reported in each category in the respective studies divided by the total number of MDR (*n* = 534) and CRAB (*n* = 468) strains. The charts were generated using GraphPad Prism v9 (GraphPad Software, San Diego, CA, USA).

Upon checking the contribution of the different LICs to either the MDR (*n* = 534) or the CRAB (*n* = 468) strains included in the current work (Figure 4A,B), the war-torn Syria had the highest proportion with 35% of the MDR and 48.7% of the CRAB. The second rank in both categories was held by Madagascar it is not one of the countries contributing with the highest number of studies (only five). However, the high percentages of both MDR and CRAB is indeed alarming and should necessitate a greater attention to the situation in this country. The situation of the rest of the countries generally reflects either the number of the studies originating from these countries or the number of strains isolated in them.

*A. baumannii* is an organism that is ubiquitous and capable of causing both hospital and community-related infections in multiple sites in the human body, yet it is predominant within immunocompromised patients [75]. Low-income countries especially those in the Sub-Sharan Africa region are already heavily affected by HIV infection together with its immunosuppressing consequences.

One aspect that is worth mentioning here is that the reason behind the low number of reports detected in our search could not be the unaffordability of publishing the results of surveillances originating from LICs in the journals indexed in the included databases in our review. This is simply because major publishers offer full waivers of publication fees for authors reporting from this group of countries, so the more likely reason is the lack of conducting the proper surveillance rather than not affording to publish its results. Additionally, publishing in the journals indexed in the searched databases could require more intensive genetic analyses of the resistance determinants and clonal relationships. The higher cost of performing these analyses, as compared to the traditional microbiological techniques, could be a barrier in front of the studies originating from LICs getting published in these indexed journals.

An alarming observation in the studies reviewed in the current work is what was noted in the island country of Madagascar. Carbapenem-resistance was detected in the country prior to the introduction of this class of antibiotics in practice in this country [21]. This highlights the role of international travel as well as the improper surveillance infrastructure in the spread of MDR and even PDR strains of formidable pathogens including *A. baumannii.* There has been previously the assumption that resistance will develop and spread in developing countries and then transfer to the developed ones through travelling. Although this could be true especially among travelers seeking refuge and asylum in the developed world. However, this should not exclude the resistance transfer in the opposite direction too. In addition, the movement of the soldiers participating in wars in the developing countries could be taking such infections back to their more developed home countries. This was clearly demonstrated in the case of *A. baumannii* infections and the American soldiers participating in the Iraq war to the extent that *A. baumannii* was then labelled as being the Iraqibacter [2].

The antimicrobial resistance burden in countries with low income is usually disproportionately higher than those with higher income. This is mainly attributed to multiple factors including the poor hygiene, the weak infrastructure of the healthcare systems, lack of treatment options, poorer understanding of transmission mechanisms, and more importantly to the widespread of infectious diseases [76]. What fuels this growing trend is that antibiotics are easily obtained as over the counter (OTC) medications and at the same time different infections are treated empirically. In addition, physicians and healthcare providers, in communities with low incomes, sometimes are under pressure to prescribe antibiotics without proper investigation of the infection causative agent [76]. Moreover, the antibiotics that are consumed in LICs could lose part of their potency during inappropriate transport or storage conditions, or due to expiration and/or adulteration [77,78]. In addition, the spread of the antimicrobial resistance in LICs could also be caused by the inappropriate use of antibiotics in conjunction with growth promoters to reduce bacterial infections to livestock that leads to the development of resistance that can be transferred to human later either directly or indirectly [79].

All these circumstances would have led to an increase in infections with MDR pathogens which are known as healthcare settings colonizers like *A. baumannii.* Despite this, we still see a very low number of reports in the literature about *A. baumannii* infections in the LICs. Alternatively, this could be due to the low level and the lack of surveillance systems within these countries mainly because of the restricted financial resources. This problem is complicated more by the fact that most of the reports coming from the LICs are based on infections detected in urban settings at large or teaching hospitals. This is due to the financial constraints among patients in rural areas so they will not seek care in hospitals, especially if this care would require travelling for long distances [80]. Accordingly, even with the scarcity of the data about infections and resistance patterns the data published about infections in LICs could be an overestimation of the real pattern in the general population. That is because most of the reported samples were collected in large hospitals in urban sites [81]. Acknowledging the lack of comprehensive population-based surveillance data, the WHO’s Global Action Plan to tackle Antimicrobial Resistance (AMR) focused on improving surveillance capacities especially in LICs through the help of multiple funders on both the governmental and private sectors [82]. Yet, there are still major challenges to overcome by these efforts such as the lack of expertise in conducting surveillances, inappropriate diagnostic capabilities regarding the equipment and/or the cold chain for transport and storage [83]. Accordingly, improving these aspects with solutions compatible with the situation in these countries is key to the success of improving the surveillance programs in LICs.

Accordingly, funding, establishing, and close monitoring of reliable antibiotic resistance surveillance systems in LICs are key steps to reach a clear picture of the situation within these countries. This should be an integrated part of comprehensive antibiotic guardianship programs that should tackle the causes of the development of the resistance and not just monitor and report it. Having such systems would enable decision and policy maker of obtaining facts that are crucial in finding solutions to contain and overcome the threats associated with the spread of several MDR pathogens not just *A. baumannii.*

The limitations of the current review could include limiting the search to the selected three databases. Accordingly, some of the relevant studies might have been missed by being part of what is known as the grey literature. Future directions could include expanding the search to other databases especially those that could include literature from countries where limited financial resources should not present an obstacle in front of reporting their findings. However, we should stress here that applying the used search criteria has resulted in selecting studies of generally high quality. Accordingly, one of the main strengths of the current review is shedding the light on this hidden danger that could be spreading in the LICs, yet it goes undocumented due to the difficulties of performing proper surveillances and reporting of the findings.

## 5. Conclusions

The tracking of the spread and extent of *A. baumannii* infections in the low-income countries is very under-represented in the literature during the first 21 years of the 21st century. Lack of proper surveillance systems capable of detecting and reporting such infections and the associated antibiotics resistance patterns is a major contributor to the scarcity of data about this part of the world. The threats of this problem could be contained by developing reliable systems that could efficiently integrate data in global surveillance programs that are continuously updated.

## Figures and Tables

**Table 1:** A summary of the studies about *A. baumannii* in LICs from 2000 to 2020.

Country	Study	Isolates (*n*)	MDR % *	CRAB%	Isolates Characterization	References
Sub-Saharan Africa						
Ethiopia	Kempf et al., 2012	40	NA	NA	*rpoB* and *recA* sequencing for genotyping	[10]
	Lema et al., 2012	5	≥20%	NA	AST with KB	[11]
	Pritsch et al., 2017	3	100%	100%	AST with KB and VITEK 2, CT102 Micro-Array, real-time PCR, WGS, MLST, and detection of the *bla*_NDM-1_	[12]
	Solomon et al., 2017	43	81%	37%	AST with KB and phenotypic detection of ESBLs and MBLs	[13]
	Bitew et al., 2017	2	100%	NA	Identification and AST with VITEK 2	[15]
	Demoz et al., 2018	1	100%	100%	AST with KB	[18]
	Gashaw et al., 2018	2	50% XDR and 50% PDR	100%	AST with KB and phenotypic detection of ESBLs and AmpC	[19]
	Moges et al., 2019	15	≥63%	Yes	AST with KB and phenotypic detection of ESBLs and carbapenemases	[14]
	Admas et al., 2020	6	100%	NA	Identification and AST with VITEK 2	[16]
	Motbainor et al., 2020	9	100%	33%	Identification with VITEK 2 and AST with KB	[17]
Madagascar	Randrianirina et al., 2010	50	≥44%	44%	AST with KB and phenotypic detection of ESBLs	[20]
	Andriamanantena et al., 2010	53	100%	100%	AST with KB and MIC determination, phenotypic detection of carbapenemases, ReP-PCR for genotyping and PCR for detection of; *bla*_AmpC_, *bla*_oxa51_, *bla*_oxa23_, *bla*_oxa24_, *bla*_VIM_, *bla*_IMP_, and *isAba-1*	[21]
	Rasamiravaka et al., 2015	10	≥50%	0%	AST with KB	[22]
	Tchuinte et al., 2019	15	100%	100%	MALDI-TOF MS for identification, AST with KB and MIC determination, WGS, MLST for genotyping and WGS detecting; *bla*_oxa51_, *bla*_oxa23_, *bla*_oxa24_, *bla*_oxa58_, and *isAba-1*	[23]
	Eremeeva et al., 2019	14	NA	NA	TaqMan PCR of the *rpoB* for identification, and PCR for detecting: *bla*_oxa51-like_, *bla*_oxa23_, *bla*_oxa24_, *bla*_VIM_, and *bla*_IMP_	[24]
Uganda	Kateete et al., 2016	40	60%	38%	AST with Phoenix Automated Microbiology System, PCR for: *bla*_oxa51-like_, *bla*_oxa51_, *bla*_oxa23_, *bla*_oxa24_, *bla*_oxa58_, *bla*_VIM_, *bla*_SPM_, and *bla*_IMP_	[26]
	Kateete et al., 2017	20	40%	35%	AST with MIC determination, PAMS, Rep-PCR for genotyping and phenotypic detection of ESBLs and AmpC	[27]
	Moore et al., 2019	3	NA	NA	qPCR TAC	[25]
	Aruhomukama et al., 2019	1077	3%	3%	AST with KB, PCR for detecting: *bla*_oxa23_, *bla*_oxa24_, *bla*_oxa58_, *bla*_VIM_, *bla*_SPM_, *bla*_KPC_, and *bla*_IMP_, phenotypic detection of carbapenemases, and conjugation to show transferability of *bla*_VIM_.	[28]
Burkina Faso	Kaboré et al., 2016	3	100%	NA	AST with KB and phenotypic detection of ESBLs	[29]
	Sanou et al., 2021 ^#^	5	100%	60%	MALDI-TOF MS for identification, AST with KB and MIC determination, phenotypic detection of ESBLs, PCR and sequencing of multiple resistance genes including; *bla*_oxa1-like_, *bla*_oxa48-like_, *bla*_NDM_, *bla*_VIM_, *bla*_SPM_, *bla*_KPC_, *bla*_CTX-M_, and *bla*_IMP_, and MLST for genotyping.	[30]
DR of the Congo	Lukuke et al., 2017	2	0%	NA	API for identification and AST with KB	[32]
	Koyo et al., 2019	15	NA	NA	qPCR and phylogenetic analysis using the *rpoB* gene	[33]
Malawi	Bedell et al., 2012	1	NA	NA	Identification with standard diagnostic techniques	[34]
	Iroh Tam et al., 2019	84	≥44%	NA	API for identification, AST with KB, and phenotypic detection of ESBLs	[35]
Mozambique	Martínez et al., 2016	1	NA	NA	16S rRNA PCR and MALDI-TOF MS for identification	[37]
	Hurtado et al., 2019	1	100%	0%	16S rRNA for identification and AST with KB	[36]
Sudan	Mohamed et al., 2019	1	NA	NA	API for identification followed by WGS	[38]
	Dirar et al., 2020	12	≥83%	89%	Identification with PAMS, AST with KB and phenotypic detection of ESBLs and carbapenemases.	[39]
Rwanda	La Scola and Raoult 2004	10	NA	NA	API for identification and *recA* genotyping	[40]
	Heiden et al., 2020	1	100%	0%	MALDI-TOF MS for identification, AST with VITEK 2, phenotypic detection of ESBLs and carbapenemases, and WGS	[41]
Burundi	La Scola and Raoult 2004	3	NA	NA	API for identification and *recA* genotyping	[40]
Mali	Doumbia-Singare et al., 2014	1	NA	NA	Not mentioned	[42]
Sierra Leone	Lakoh et al., 2020	14	≥40%	10%	Identification and AST with VITEK 2	[43]
Somalia	Mohamed et al., 2020	7	100%	100%	AST with KB	[44]
Niger	Louni et al., 2018	29	NA	NA	qPCR and *rpoB* PCR for identification and phylogenetic analysis	[45]
Central African Republic	No Reports					
Chad	No Reports					
Eritrea	No Reports					
Gambia	No Reports					
Guinea	No Reports					
Guinea-Bissau	No Reports					
Liberia	No Reports					
South Sudan	No Reports					
Togo	No Reports					
Middle East and North Africa						
Syria	Hamzeh et al., 2012	260	≥65%	65%	Identification and AST with PAMS	[46]
	Teicher et al., 2014	6	100%	80%	API for identification and AST with MicroScan Walk-Away System	[47]
	Peretz et al., 2014	5	100%	NA	Not mentioned	[50]
	Rafei et al., 2014	4	100%	100%	Identification with *rpoB* sequencing and *bla*_oxa51_, PCR, AST with KB and Etest, PCR for: *bla*_oxa23-like_, *bla*_oxa24-like_, *bla*_oxa58-like_, and *bla*_NDM_, and PFGE for genotyping	[53]
	Heydari et al., 2015	1	100%	100%	Identification and AST with VITEK 2, phenotypic detection of ESBLs and carbapenemases, PCR for the *bla*_NDM_, PFGE and MLST for typing	[51]
	Rafei et al., 2015	59	Yes	74%	Identification with MALDI-TOF MS, *rpoB* sequencing and *bla*_oxa51_ PCR, AST with KB and Etest, PCR for detecting: *bla*_oxa23_, *bla*_oxa24_, *bla*_oxa58_, *bla*_NDM-1_, *bla*_VIM_, *bla*_oxa143_, and *bla*_IMP_, and MLST for typing	[54]
	Herard and Fakhri 2017	38	NA	NA	Not mentioned	[48]
	Salloum et al., 2018	2	100%	100%	AST with KB and Etest, PCR for *bla*_oxa58_ and *bla*_NDM_, plasmid typing with PBRT, MLST, and WGS	[55]
	Fily et al., 2019	6	NA	67%	AST with KB	[49]
	Hasde et al., 2019	5	NA	NA	Not mentioned	[52]
Yemen	Bakour et al., 2014	3	100%	100%	API and MALDI-TOF MS for identification, AST with KB and E-test, phenotypic detection of carbapenemases, PCR detection of: *bla*_oxa23_, *bla*_oxa24_, *bla*_oxa58_, *bla*_NDM_, *bla*_VIM_, *bla*_SIM_, *bla*_oxa48-like_, *bla*_IMP_ and others, and MLST	[56]
	Fily et al., 2019	1	NA	100%	AST with KB	[49]
South Asia						
Afghanistan	Sutter et al., 2011	57 ^¥^	≥75%	76%	Identification and AST with MicroScan autoSCAN-4	[57]
Latin America and The Caribbean						
Haiti	Potron et al., 2011	3	66.7%	0%	API and 16sRNA for identification, AST with KB and E-test, phenotypic detection of ESBLs, PCR for detection of: *bla*_TEM_, *bla*_SHV_, *bla*_PER-1_, *bla*_VEB-1_, *bla*_GES-1_, and *bla*_CTX-M_	[58]
	Marra et al., 2012	1	100%	0%	Identification and AST with VITEK 2	[59]
	Murphy et al., 2016	4	≥25%	25%	AST but the method was not indicated	[60]
	Chaintarli et al., 2018	2	0%	0%	Identification and AST with VITEK 2 and phenotypic detection of ESBLs	[61]
	Roy et al., 2018	0 ^ϕ^	NA	NA	Metagenomic analyses of water samples	[62]
Europe and Central Asia						
Tajikistan	No Reports					
East Asia and Pacific						
Democratic People’s Republic of Korea	No Reports					

Abbreviations: API: Analytical Profile Index, AST: Antibiotic Susceptibility testing, CRAB: Carbapenem-resistant *A. baumannii*, KB: Kirby-Bauer disc diffusion method, MALDI-TOF MS: Matrix-Assisted Laser Desorption/Ionization Time-of-Flight Mass Spectrometry, MDR: Multi-drug resistant, MLST: Multilocus sequence typing, NA: not available, PAMS; Phoenix Automated Microbiology System, PDR: Pan-drug-resistant, PBRT: PCR-based replicon typing, PCR: Polymerase chain reaction, PFGE: Pulse Field Gel Electrophoresis, rep-PCR: Repetitive element sequence-based PCR, WGS: Whole Genome Shotgun, TAC: TaqMan Array Card, XDR: Extensively drug-resistant. * When the MDR% is not directly mentioned, it was presented as ≥lowest % of resistance among the tested antibiotics classes. All %s were approximated to the nearest whole number. ^#^ The study was originally published in Jun 2020, then appeared in its final version in Jan 2021. ^¥^ *Acinetobacter* spp. Including *A. baumannii*. ^ϕ^ No isolates were obtained but *A. baumannii* DNA was detected in the samples.

## Data Availability

Not applicable.
